# Anomalous vascularization in a Wnt medulloblastoma: a case report

**DOI:** 10.1186/s12883-016-0632-1

**Published:** 2016-07-15

**Authors:** Angela Di Giannatale, Andrea Carai, Antonella Cacchione, Antonio Marrazzo, Vito Andrea Dell’Anna, Giovanna Stefania Colafati, Francesca Diomedi-Camassei, Evelina Miele, Agnese Po, Elisabetta Ferretti, Franco Locatelli, Angela Mastronuzzi

**Affiliations:** Department of Hematology/Oncology and Stem Cell Transplantation, Bambino Gesù Children’s Hospital, IRCCS, Piazza di Sant’Onofrio, 4 – 00165 Rome, Italy; Department of Neuroscience and Neurorehabilitation, Neurosurgery Unit, Bambino Gesù Children’s Hospital, IRCCS, Piazza di Sant’Onofrio, 4 – 00165 Rome, Italy; Imaging Department, Neuroradiology Unit, Bambino Gesù Children’s Hospital, IRCCS, Piazza di Sant’Onofrio, 4 – 00165 Rome, Italy; Department of Laboratories - Pathology Unit, Bambino Gesù Children’s Hospital, IRCCS, Piazza di Sant’Onofrio, 4 – 00165 Rome, Italy; Center for Life NanoScience@Sapienza, Istituto Italiano di Tecnologia, Viale Regina Elena 291, 00161 Rome, Italy; Department of Molecular Medicine, Sapienza University, Viale Regina Elena 291, 00161 Rome, Italy; Department of Pediatric Science, University of Pavia, Viale Brambilla 74, 27100 Pavia, Italy

**Keywords:** Case report, Medulloblastoma, Wnt/β-catenin, Angiogenesis

## Abstract

**Background:**

Medulloblastoma is the most common malignant brain tumor in children. To date only few cases of medulloblastoma with hemorrhages have been reported in the literature. Although some studies speculate on the pathogenesis of this anomalous increased vascularization in medulloblastoma, the specific mechanism is still far from clearly understood. A correlation between molecular medulloblastoma subgroups and hemorrhagic features has not been reported, although recent preliminary studies described that WNT-subtype tumors display increased vascularization and hemorrhaging.

**Case presentation:**

Herein, we describe a child with a Wnt-medulloblastoma presenting as cerebellar-vermian hemorrhagic lesion. Brain magnetic resonance imaging (MRI) showed the presence of a midline posterior fossa mass with a cystic hemorrhagic component. The differential diagnosis based on imaging included cavernous hemangioma, arteriovenous malformation and traumatic lesion. At surgery, the tumor appeared richly vascularized as documented by the preoperative angiography.

**Conclusions:**

The case we present showed that Wnt medulloblastoma may be associated with anomalous vascularization. Further studies are needed to elucidate if there is a link between the hypervascularization and the Wnt/β-catenin signaling activation and if this abnormal vasculature might influence drug penetration contributing to good prognosis of this medulloblastoma subgroup.

## Background

Medulloblastoma is the most common malignant brain tumor in children, representing approximately 25 % of all pediatric brain tumors [1]. Current molecular stratification consists in four subgroups associated with different pathways defined as Wnt, Sonic Hedgehog Homolog (SHH), group 3, and group 4 [2]. Wnt subtype is the rarest subgroup, accounting for 10 % of medulloblastomas [3] and patients with this pathway activation have a very good long-term prognosis [4]. However, the biological effect of Wnt/β-catenin signaling activation and the link with a better prognosis has not been clarified yet. Few cases of medulloblastoma presenting with spontaneous hemorrhage are reported in the literature [5]. Although some studies speculate on the etiology of the anomalous increased vascularization in medulloblastoma [6], a clear pathogenetic role has not been identified. A correlation between specific medulloblastoma subgroup and hemorrhagic features has not been reported [7], however a recent paper demonstrated that Wnt medulloblastoma secretes Wnt antagonists that increase the permeability of the blood–brain barrier (BBB) [8]. Furthermore, a personal communication hypothesized that this aberrant vascular network may be associate with the Wnt subgroup [9]. Herein, we report the case of a child with a Wnt medulloblastoma presenting as a hemorrhagic cerebellar-vermian lesion.

## Case presentation

**Fig. 1:**
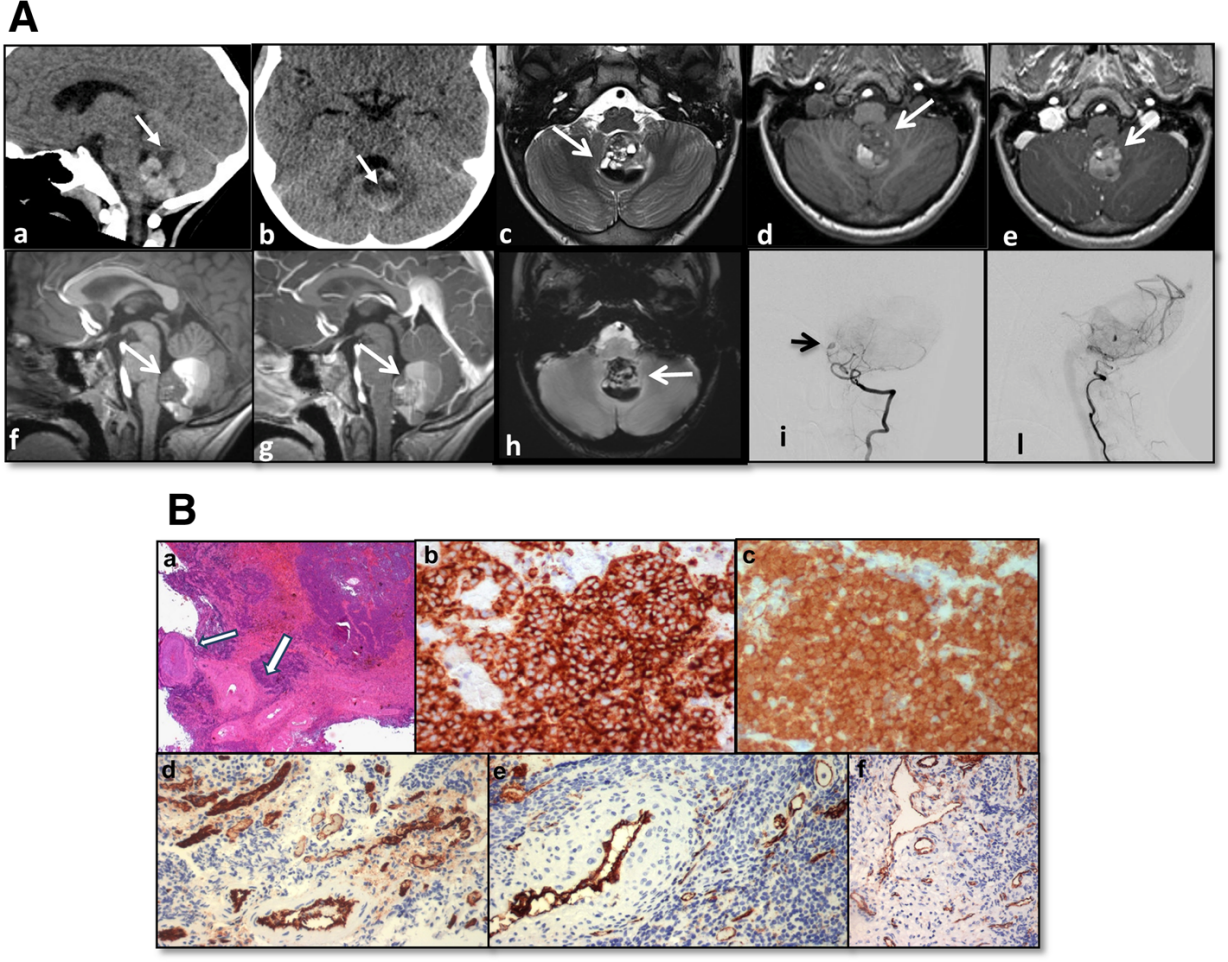
MRI and histological findings in the hemorrhagic lesion. (**A**) [CT and MRI findings in the hemorrhagic cerebellar lesion]. Sagittal (a) and Axial (b) MDCT reconstruction images show a hyperdense cerebellar-vermian lesion, with fluid-blood levels (white arrows), confirmed by MRI scan. Axial T2-weighted MRI (c) showed a hemorrhagic cerebellar-vermian lesion, expanding into IV ventricle, with multiple fluid-blood levels (white arrow). Axial-Sagittal T1-weighted MRI, without (d, e) and with (f, g) gadolinium, revealed a solid component inhomogeneously contrast-enhanced (white arrows). Axial gradient-echo sequence (h) showed lack of hypointense hemosiderin rim (cavernous hemangioma classical finding, white arrow). Preoperative left vertebral angiograms (i, l) show hypoplastic vertebral artery terminating as posterior inferior cerebellar artery (vascular variation) and the tumor stain; an aneurysm-like formation (arrow) is seen in the arterial phase. The tumor is fed by the vermian branch of the left posterior inferior cerebellar artery. (**B**) [Histological findings in hemorrhagic medulloblastoma]. Proliferation of small undifferentiated cells showing a diffuse/multinodular pattern (middle-right), associated to anomalous, thick-walled vascular structures (arrows) (a, H&E, 2.5x). Cells were synaptophysin (b, 20x) and beta-catenin (both cytoplasm and nucleus) positive (c, 20x). Anomalous vascularization was characterized by clusters of anomalous, thick-walled arterial-type vessels (d and f, CD31 20x) along with numerous variably anastomosing small venous and capillary structures (e, CD31, 20x)

**Fig. 2:**
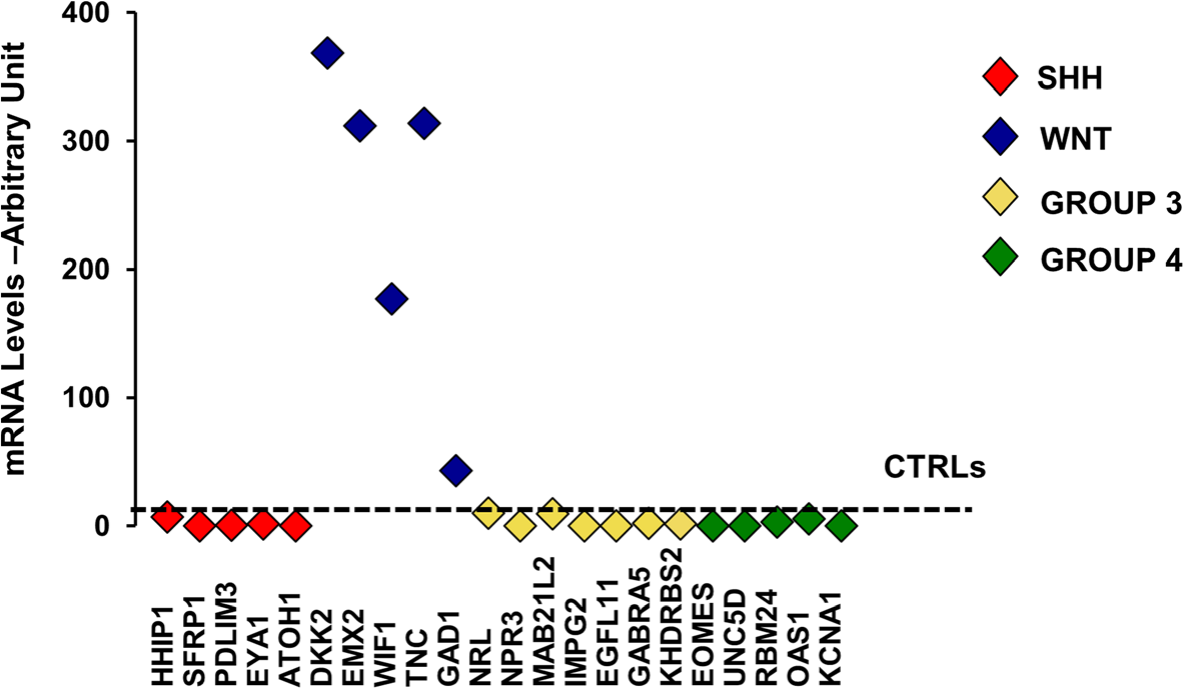
Molecular characterization of medulloblastoma. mRNA levels of the indicated genes in medulloblastoma are compared to normal cerebellum as control (CTRLs). Genes are grouped into four molecular subgroups (WNT, SHH, GROUP 3, GROUP 4) as indicated by the four different colors. Relative quantification values are expressed as linear scale arbitrary units

A 7-year-old girl presented to the emergency department of Bambino Gesù Children’s Hospital with a one-month history of headache and vomiting. Neurological examination was normal apart from mild dysmetria at the upper extremities. Multidetector computed tomography (MDCT) reconstruction images show a hyperdense cerebellar-vermian lesion, with fluid-blood levels. This finding was confirmed by brain magnetic resonance imaging (MRI) that showed the presence of a mass with a hyperintense cystic hemorrhagic component, heterogeneous enhancement, poor perilesional edema and absence of obstructive hydrocephalus (Fig. 1A). Spinal MRI and cerebrospinal fluid study were normal. Due to these atypical features a cerebral angiography was performed, showing an intratumoral aneurysm-like formation supplied by a vermian branch of the left posterior inferior cerebellar artery (Fig. 1A). She underwent a midline suboccipital craniotomy with complete resection of the vermian tumor. At surgery, the tumor appeared richly vascularized from vermian branches as documented by the preoperative angiography. Histology (Fig. 1B) showed a diffuse and multinodular proliferation of small undifferentiated cells and immunohistochemistry revealed positivity for synaptophysin and β-catenin (both cytoplasmic and nuclear), all features consistent with the diagnosis of classic medulloblastoma. The tumor was characterized by anomalous vascularization and harbored some clusters of anomalous, thick-walled vascular structures along with numerous variably anastomosing small venous and capillary structures. Gene expression profile of the tumor confirmed a Wnt molecular subgroup (Fig. 2). C-Myc amplification was negative (C-Myc/SPAST and C-Myc/PI4KA). She started therapy as per standard arm of European HIT-SIOP PNET 4 trial which is used for the treatment of standard risk medulloblastoma. According to this protocol, she received radiotherapy (23 Gy to the craniospinal axis and a total of 54 Gy to posterior fossa) given concurrently with weekly vincristine (1.5 mg/m^2^ i.v.). This treatment was followed by 8 cycles of maintenance-chemotherapy consisting of cisplatin (70 mg/m^2^ i.v.) and lomustine (75 mg/ m^2^ orally) on day 1 associated to vincristine (1.5 mg/m^2^ i.v.) on day 1, 8 and 15. At 22 months after diagnosis she was doing well with no evidence of disease.

The consensus held in Boston in 2010 supported the existence of four main medulloblastoma subgroups based on the molecular profiling and provided important insights not only in the selection of patients for molecular targeted therapies but also in the outcome prediction [2, 3, 10]. Medulloblastomas with activation of Wnt/β-catenin pathway are rarely metastatic and appear to be a less aggressive variant associated with an excellent prognosis [11]. However, the significance of Wnt activation in medulloblastoma remains to be determined. Several findings suggest that wild type β-catenin has an important physiological role in CNS angiogenesis. During embryogenesis, the Wnt pathway has direct actions on axonal growth through interaction with β-catenin complex [12]. Moreover, it has been demonstrated that Wnt signaling plays an active role in the induction and maintenance of BBB characteristics during embryonic and postnatal development particularly by regulating tight junction proteins expression [13]. Indeed, Wnt/β-catenin signaling is very important for central nervous system (CNS) angiogenesis and conditionally inactivation of β-catenin in the endothelium has been described to alter the development of head vasculature resulting in early embryonic lethality of mice [14, 15]. β-catenin–null animals show vessel fragility, in association to a decrease in intercellular adhesion strength and an increase in paracellular permeability leading to vascular leakage and frequent hemorrhages. The critical role of canonical Wnt signaling in endothelial cells for formation and differentiation of the CNS vasculature has been described also in genetic mouse models [16]. Recently has been reported that G-protein coupled receptor 124 (GPR124) functions as a specific co-stimulator of β-catenin signaling in brain endothelium and its disruption led to defective CNS angiogenesis and blood brain barriergenesis in mice [17]. In mouse embryos, eliminating neuroepithelial Wnt7a and Wnt7b, or endothelial Gpr124 or β-catenin, leads to a reduced CNS angiogenesis with formation of abnormal vascular structures [13, 16, 18] and has been demonstrated that the interaction of Gpr124 with Reck strongly synergize to promote Wnt/β-catenin signaling during brain angiogenesis [19].

Nevertheless, most of the studies correlating Wnt signaling with CNS vasculature anomalies are developmental phenotypes, and the relevance to post-natal development is not clear. Moreover, β-catenin is important in the regulation of vascular endothelial cell-cell adhesions and barrier function by linking the VE-cadherin junction complex to the cytoskeleton and thus vascular anomalies may arise for a non-signaling role [20].

A recent study showed that genetically modified mouse models harboring Wnt-medulloblastoma had more hemorrhagic tumors compared to SHH or group 3 tumors [8]. The authors demonstrated that these effects occur postnatally and reveal that Wnt medulloblastoma secretes Wnt antagonists increasing the permeability of the BBB [8].

We report a girl with a Wnt medulloblastoma presenting an anomalous vascularization. Differential diagnosis based on MRI imaging appearance includes cavernous hemangioma, arteriovenous malformation and traumatic lesion. Angiographic opacification of dilated arteriolar vessels with slow flow and lack of early venous drainage confirmed an aberrant tumor vascular network. This patient is doing well at 22 months after diagnosis. Medulloblastoma can present heterogeneous features on MRI with variable enhancement patterns, cystic areas, hemorrhage and calcification [21]. Although intratumoral bleeding can be found in brain tumors, only few cases of medulloblastoma with spontaneous hemorrhages have been reported in the literature [5, 22]. Park et al. showed that the incidence of spontaneous hemorrhage was 5.6 % in patients with primary or recurrent medulloblastoma [23]. A study evaluating vascular regulatory expression profiles across medulloblastoma subgroups documented an upregulation of proangiogenic factors in SHH subgroup [6]. Despite specific mechanisms driving aberrant vascularization and hemorrhage in medulloblastoma remain not completely elucidated, new researches revealed that medulloblastoma genotype dictates tumor vessels phenotype [8].

## Conclusions

We reported a case of Wnt/β-catenin medulloblastoma associated with an anomalous vascularization. This may support the evidence that Wnt medulloblastomas may be associated to aberrant vascular network contributing to a better drug penetration and therefore to their excellent prognosis [8]. This finding should be further investigated in a large patient cohort in order to elucidate the vascular microenvironment in Wnt medulloblastoma subgroup.

## Abbreviations

SHH, sonic hedgehog homolog; MDTC, multidetector computed tomography; MRI, magnetic resonance imaging; BBB, blood–brain barrier; CNS, central nervous system
